# A Cross-Sectional Study Among Medical Residents with Noninvasive Rapid Serological Test for *Helicobacter Pylori*

**DOI:** 10.4103/0974-777X.68544

**Published:** 2010

**Authors:** Mohanakrishnan

**Affiliations:** *Department of Microbiology, Meenakshi Medical College & Research Institue, Enathur, Kanchipuram, India*

Sir,

Helicobacter pylori infection is causally associated with peptic ulcer disease and gastric carcinoma. The goal of this study was to determine the seroprevalence rate of *Helicobacter pylori* (Hp) antibodies in residents, and its relationship with dyspepsia. This cross-sectional study was carried out in September 2009 on a population of 55 residents who were willing to participate in this study at Meenakshi Medical College. The study was carried out after obtaining proper approval from the ethical committee. Each participant was interviewed using a questionnaire.

Rapid serological test for H. pylori antibody (ACCUC80305503/2010) using immunochromatography was carried out. The kit has >99% sensitivity, 86.7% specificity, 93.2% accuracy and 95% confidence intervals. After obtaining informed consent, 3 mL of venous blood was taken from the medical students for serological rapid test for *H. pylori.*

In this cross-sectional study, only 55 students could be screened for *H. pylori* serology. There were 18 (32.72%) male and 37 (67.27%) female students with or without specific gastrointestinal signs and symptoms, like heartburn, regurgitation, abdominal pain, vomiting and hemetemesis/ melena, of whom 10 (18.18%) were positive for *H. pylori* IgG antibodies. The age of the participants was between 21 and 23 years, and the mean age was 22. At the end of this study, 6 (10.91%) males and 4 (7.27%) females were positive for *H. pylori* IgG tests (ACCUC803055). Finally, the *H. pylori* positivity rate was 33.33% (6/18) among males and 10.81% (4/37) among females. Of the 21 (38.18%) participants reporting dyspeptic symptoms, 4 (2 males, 2 females) (19%) were *Hp* positive. Of the 34 asymptomatic students, 6 (4 males, 2 females) (17.64%) were *Hp* positive. Statistical analysis did not show a significant difference in the frequency of *Hp* infection between asymptomatic students and students reporting dyspeptic symptoms.

This study should be followed by a large-scale study with multicentric screening to validate the results, as the sample size of this study was small because of the unwillingness of the medical students to participate and cost constraints.

According to the study, the prevalence rate was 18.88% for *H. pylori* in residents, which is comparatively low than that reported in a Brazil study by Melo ET *et al*,[1] in which 38.4% positivity was observed. The male group of medical students showed high positivity rate, viz., 33.33%, probably due to unhealthy life style, like frequent intake of spicy food, irregular diet and inadequate sleep. There was no significant difference in Hp prevalence between dyspeptic students and asymptomatic students.[2] Hence it can be concluded that according to this study, the *H. pylori* infection is common in males and does not have significant relationship with dyspepsia.
